# Effect of vegetarian diets on renal function in patients with chronic kidney disease under non-dialysis treatment: A scoping review

**DOI:** 10.1590/2175-8239-JBN-2021-0126

**Published:** 2022-01-31

**Authors:** Agnes Valim, Larissa Salomoni Carpes, Bruna Bellincanta Nicoletto

**Affiliations:** 1 Universidade de Caxias do Sul, Curso de Nutrição, Caxias do Sul, RS, Brasil.; 2 Universidade Federal do Rio Grande do Sul, Faculdade de Medicina, Programa de Graduação em Alimentação, Nutrição e Saúde, Porto Alegre, RS, Brasil.; 3 Universidade de Caxias do Sul, Curso de Nutrição, Área de Conhecimento em Ciências da Vida, Caxias do Sul, RS, Brasil.

**Keywords:** Diet, Vegetarian, Renal Insufficiency, Chronic, Conservative Treatment, Dieta Vegetariana, Insuficiência Renal Crônica, Tratamento Conservador

## Abstract

Vegetable protein diets (VPDs) in chronic kidney disease (CKD) patients may be related to beneficial biological actions and possibly clinical impact. This is a scoping review that merge studies that evaluated the effect of a vegetarian diet on kidney function in adults with CKD under non-dialysis treatment. The evaluated outcome was the impact in renal function assessed by eGFR or creatinine clearance. MEDLINE (accessed by PubMed) was searched up to September 8, 2020. Data were extracted by two independent reviewers, who also assessed the quality of the studies. Of 341 retrieved articles, 4 studies assessing 324 patients were included in the analysis. One study showed that a very low-protein ketoanalogue-supplemented vegetarian diet had benefits in relation to a conventional low-protein diet, while the other three studies demonstrated no difference in kidney function between the evaluated diets. Additional studies are needed to assess the benefits of vegetarian diets for further recommendations in CKD management.

## Resumo

As dietas de proteína vegetal (VPDs, do inglês *vegetable protein diets*) em pacientes com doença renal crônica (DRC) podem estar relacionadas a ações biológicas benéficas e possivelmente ao impacto clínico. Esta é uma revisão de escopo que reúne estudos que avaliaram o efeito de uma dieta vegetariana na função renal em adultos com DRC sob tratamento não-dialítico. O desfecho analisado foi o impacto na função renal avaliado pela TFGe ou pelo clearance de creatinina. O MEDLINE (acessado via PubMed) foi pesquisado até 8 de Setembro de 2020. Os dados foram extraídos por dois revisores independentes, que também avaliaram a qualidade dos estudos. De 341 artigos recuperados, foram incluídos na análise 4 estudos avaliando 324 pacientes. Um estudo mostrou que uma dieta vegetariana hipoproteica suplementada com cetoanálogos teve benefícios em relação a uma dieta hipoproteica convencional, enquanto os outros três estudos não demonstraram diferença na função renal entre as dietas avaliadas. São necessários estudos adicionais a fim de avaliar os benefícios de dietas vegetarianas para maiores recomendações no manejo da DRC.

## Introduction

Chronic kidney disease (CKD) is defined by a decreased renal function, that is, a glomerular filtration rate (GFR) of less than 60 mL/min per 1.73 m^2^, or markers of kidney damage, or both, for at least 3 months, regardless of the underlying cause. Diabetes and hypertension are the main causes of CKD^1^. Simple blood and urine tests can detect CKD and low-cost treatments can slow disease progression, reduce the risk of stroke and heart attacks, and improve quality of life^2^.

Dietary management is a recognized treatment for CKD. The National Kidney Foundation recommends protein restriction with or without keto acid analogs for adults with CKD 3-5 without diabetes who are metabolically stable and under close clinical supervision, to reduce the risk for end-stage kidney disease (ESKD) and death and improve their quality of life^3^. For these patients, the protein intake level can be safely decreased to 0.55 to 0.6 g protein/kg per day^3^. If necessary, a further reduction in protein intake to 0.3 to 0.4 g protein/kg per day can be achieved with the addition of ketoacid analogues to ensure a sufficient balance of essential amino acids. In adult diabetic patients with CKD 3-5, a dietary protein intake of 0.6-0.8 g/kg body weight per day is recommended to maintain a stable nutritional status and optimize glycemic control^3^.

The protein source may also be relevant in the management of CKD^4^. Vegetable protein diets (VPDs) in CKD patients may have positive biological actions and clinical benefits through some suggested mechanisms. There is evidence that VPDs could reduce the expression of renin-angiotensin^5^ and decrease CKD development and progression, presumably through favorable effects on GFR^6^. VPDs are also associated with decrease in serum phosphate and fibroblast growth factor 23 levels in CKD patients not receiving dialysis and reduction of uremic toxins^7^, inflammation^8^, and hypertension^9^. VPDs could then be used to reduce phosphorus load and potentially CKD progression in these patients^3^. Moreover, increased intake of plant rather than animal sources of protein could also reduce acid load and metabolic acidosis^4^, which would have a positive impact on disease management. Therefore, the objective of this review is to merge studies that evaluate the effect of a vegetarian diet on kidney function in adults with CKD under non-dialysis treatment.

## Methodology

### Eligibility criteria and search strategy

All relevant articles, regardless of language, were identified by searching MEDLINE (accessed by PubMed) up to September 8, 2020. The MEDLINE search strategy was as follows: (vegetarian OR vegetarian diet OR plant based OR plant based diet OR vegetarianism) AND (chronic kidney disease OR chronic renal disease OR renal insufficiency OR kidney insufficiency). All potentially eligible studies were considered for this scoping review.

### Eligibility criteria

Studies comparing a vegetarian to a non-vegetarian diet in adults with CKD in non-dialysis treatment were included. The evaluated outcome was the impact on renal function assessed by eGFR (mL/min/1.73 m^2^) or creatinine clearance (mL/min). Review and experimental studies were excluded. A study that evaluated pregnant women was also excluded because it involved a population with different characteristics and needs.

Two investigators (A.V.V. and L.S.C) independently evaluated retrieved articles. First, titles and abstracts were assessed. If the abstract did not provide sufficient information regarding eligibility criteria, the full text of the article was evaluated. Reviewers were not blinded to the authors, institutions, or article journals. The same investigators independently conducted data extraction. Disagreements were resolved by consensus or by a third reviewer (B.B.N.).

The following study characteristics were extracted: author’s name, year of publication, study design and objective, intervention and control groups, follow-up length, stage of CKD, eGFR (mL/min/1.73 m^2^) or creatinine clearance (mL/min), age (years), sex, body mass index (BMI) and other outcomes and relevant results.

### Quality assessment

Risk of bias was independently assessed by two authors (A.V.V. and L.S.C) on the domains: selection bias (random sequence generation, allocation concealment), performance bias (blinding of participants and personnel), detection bias (blinding of outcome assessment), attrition bias (incomplete outcome data), reporting bias (selective reporting), and other biases using the Cochrane risk of bias tool^10^. All domains were scored as (1) low risk of bias, (2) unclear, or (3) high risk of bias. Disagreements were solved by consensus or by a third reviewer (B.B.N).

## Results

**Figure 1:**
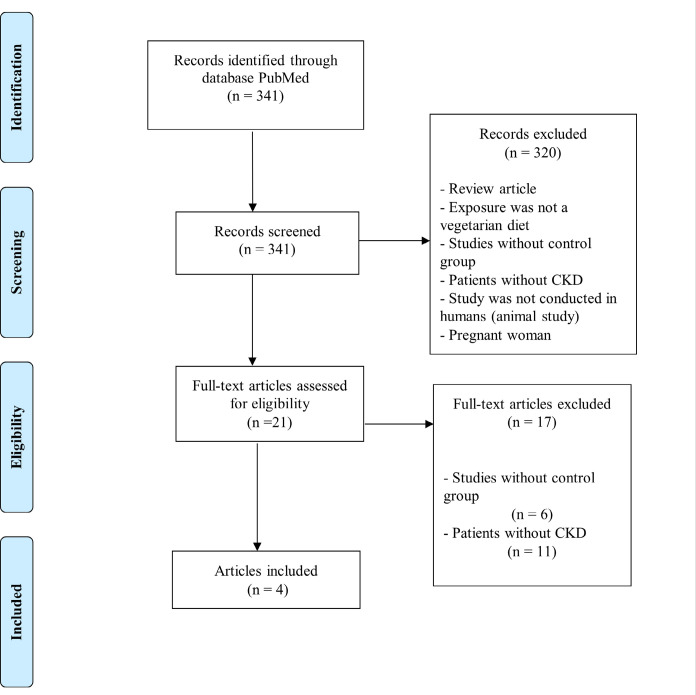
Flow diagram of study selection.

The literature search resulted in 341 potentially relevant articles. Of these, 329 were excluded based on title and abstract, and 21 studies were assessed for full- text evaluation. After eligibility criteria application, four studies were included in this scoping review **(** Figure 1 **)**.

### Study characteristics

The studies’ characteristics are presented in Table 1. The year of publication ranged from 1998 to 2018. Of the 4 studies included in our analysis, 1 was a randomized controlled trial^11^, 2 were crossover studies^12,13^, and 1 had a cross-sectional design^14^. The sample sizes varied from 8 to 207 patients.

**Table 1:** Characteristics of the studies included in the review

Study (year)	Design	Objective	Intervention and control groups	Follow-up length	Stage of CKD	eGFR or creatinine clearance	Age (years)	Sex	BMI (kg/m^2^)	Outcomes and results
Chang et al. (2018). Taiwan.	Cross- sectional study.	To investigate the effects of different proportions and sources of protein in lacto- ovo vegetarian and omnivorous diets, as well as the influence of adequate dietary protein intake, on renal function and nutritional status of Taiwanese patients.	I: 40 (lacto- ovo vegetarians)	Outpatients who visited the Department of Nephrology and Nutrition at Chung Shan Medical University Hospital between June 2011 and May 2015 were enrolled.	Stages 3 to 5.	eGFR: 21.7 ± 12.1 mL/min/1.73 m^2^	68.3 ± 11.9	Male: 37.5% Female: 2.5%	23.3 ± 3.5	The indicators of renal function including eGFR were not significantly different between lacto-ovo vegetarian and omnivorous patients with CKD (p= 0.305).
			C: 60 (omnivores)			eGFR: 24.6 ± 14.9 mL/min/1.73 m^2^	64.4 ± 13.7	Male: 55% Female: 45%	24.9 ± 3.7	
Garneata et al. (2016). Romênia.	Prospective, randomized, controlled trial.	To evaluate safety and efficacy of very low-protein ketoanalogue-supplemented vegetarian diet (KD) compared with conventional low-protein diet (LPD).	I: 104 (KD)	15 months	Stages 4 and 5.	eGFR: Baseline: 18.0 (15.5 to 20.1) mL/min/1.73m2 End of study: 15.1 (13.2 to 17.4) mL/min/1.73m^2^	55.2	Male: 63% Female: 37%	23.6 (23.1 to 24.2)	A significant lower percentage of patients in the KD group reached the primary end point (RRT initiation or a >50% reduction in the initial eGFR): 13% versus 42% in the LPD group (P<0.001). The difference between arms was >10%. RRT initiation was required in a lower proportion in the KD group (11% versus 30%; P<0.001).
			C: 103 (LPD)			eGFR: Baseline: 17.9 (14.3 to 19.3) mL/min/1.73m^2^ End of study: 10.8 (9.0 to 12.2) mL/min/1.73m^2^	53.6	Male: 59% Female: 41%	23.2 (22.7 to 23.7)	
Moe et al. (2010) Estados Unidos.	Crossover trial.	To determine if the dietary protein source of phosphate influences phosphorus metabolism and hormonal changes in humans because this would affect dietary recommendations.	I: 8 (Vegetarian diet) C: 8 (Meat diet)	7 days	Stage late 3 or stage 4.	CCr Before intervention: 43 ± 11 mL/min After intervention: 44 ± 16 mL/min CCr Before intervention: 47 ± 16 mL/min After intervention: 47 ± 16 mL/min	61 ± 8.4 61 ± 8.4	Male: 4 Female: 4 Male: 4 Female: 4	32 ± 5 32 ± 5	There was no difference in creatinine between the two diets (P: NS).
Soroka et al. (1998) Israel.	Randomized crossover design.	To compare the effect of a soya-based vegetarian low-protein diet (VPD) and an animal-based low- protein diet in patients with moderate to severe CKD.	I: 9 (vegetarian low-protein diet) C: 9 (animal- based low- protein diet)	6 months	The creatinine clearance (CCr) had to be between 15 and 50 mL/min/1.73 m^2^ and the 24- hour urinary protein excretion <3 g/day.	CCr: 25.09 ± 2.9 mL/min/1.73 m^2^ (end of 6 months) CCr: 28.62 ± 4.0 mL/min/1.73 m^2^ (end of 6 months)	30-85 30-85	Male: 5 Female: 4 Male: 5 Female: 4	NA NA	There were no significant differences between the groups (P > 0.05).

CKD: Chronic kidney disease; I: Intervention; C: Control; eGFR: estimated glomerular filtration rate; KD: very low-protein ketoanalogue-supplemented vegetarian diet; LPD: conventional low-protein diet; NS: not significant; CCr: creatinine clearance; RRT: renal replacement therapy.

One of the studies^11^ showed that a very low-protein ketoanalogue-supplemented vegetarian diet had benefits in relation to a conventional low-protein diet. After 15 months of follow-up, 13% of patients in the vegetarian diet group reached the primary end-point (renal replacement therapy initiation or a >50% reduction in the initial eGFR) versus 42% in the low-protein diet group (P<0.001). Renal replacement therapy initiation was less required in the vegetarian diet group (11% versus 30%; P<0.001). After adjustment for the other significant outcome predictor (eGFR, body mass index, C-reactive protein, and angiotensin-converting enzyme inhibitor/angiotensin receptor blocker therapy), the very low-protein ketoanalogue-supplemented vegetarian diet remained associated to a lower probability of reaching the end-point^11^. Moreover, the vegetarian diet was also associated to metabolic improvements in this study^11^.

Three of the studies^12-14^ demonstrated that there is no difference in kidney function, as measured by eGFR (mL/min/1.73 m^2^)or creatinine clearance (mL/min), between a vegetarian diet and a meat-based diet **(** Table 1 **).**

### Risk of bias across studies

One of the studies^14^ was not evaluated by the Cochrane risk of bias tool^5^ as it was a cross-sectional study, which does not allow a reliable assessment of the outcome. The others studies^11,12,13^ were classified as high risk of performance and detection biases (the groups were aware of the intervention they were receiving - change in diet) and low risk of selection, attrition, reporting and other biases.

## Discussion

The results of this review indicate that it is likely that a very low-protein ketoanalogue-supplemented vegetarian diet could have benefits in patients with advanced CKD^11^. However, only one of the evaluated studies proved this association, while the other three showed no significant difference in renal function between the vegetarian and the non-vegetarian diets^12-14^.

Some metabolites that are responsible for adverse outcomes in CKD could be reduced in patients who adhere to a vegetarian diet, having a positive impact in disease management^15^. In end-stage kidney disease, the accumulation of uremic toxins, such as indoxyl sulfate (IS), indole-3 acetic acid, p-cresyl sulfate (PCS), and trimethylamine N-oxide (TMAO), is related to CKD progression and mortality^13^. These metabolites are products of amino acids and amines breakdown that are not excreted by the damaged kidney. Vegetarian diets have lower contents of lecithin, choline, and l-carnitine, which might result in a lower production of TMAO. Also, a vegetarian diet with a lower protein/fiber index could reduce the PCS and IS levels^16^.

Exposure to uremic toxins can also affect the microbiota. A dysbiotic gut microbiome in CKD favors pathobionts overgrow, such as bacteria that produce uremic toxins. In dysbiosis, the gut barrier permeability is increased, which is associated to systemic inflammation, adverse cardiovascular outcomes, and CKD progression^19^. A fiber-rich diet, such as a vegetarian/vegan diet, can provide a healthy gut microbiota and may improve the dysbiosis associated to CKD. Consequently, the systemic inflammation and oxidative stress in patients who adhere to this diet could be reduced^18,19^.

Another mechanism has been suggested for the benefits associated to a vegetarian diet. A study including renal transplant patients showed that a soy protein-based diet for 5 weeks improved endothelial function, mediated by an increase in the L- arginine/asymmetric dimethyl arginine (ADMA) ratio, independently of change in lipid profile, oxidative stress, or isoflavones^20^.

CKD progression could also be related to the diet phosphorus content, which is low in vegetarian diets. This can be explained by the fact that this type of diet is practically free of phosphorus-containing additives, which are widely used in processed foods such as meat and poultry^21^. Compared to phosphorus from unprocessed foods, phosphorus in additives has a higher bioavailability, as it is almost completely absorbed by the intestinal tract^17,22^. Although this review was not designed to evaluate phosphorus levels, it is important to mention that one of the included studies demonstrated that only 1 week of a vegetarian diet led to lower serum phosphorus and FGF-23 levels^12^. This association also exists with diets not strictly vegetarian but with a higher percentage of plant-based proteins^23^.

Some limitations were identified in the evaluated studies. First, the cross-sectional design of the Chang et al. (2018) study^14^ does not allow for a cause and effect association. The study of Moe et al. (2010)^12^ allows a more reliable assessment of the intervention as there was a washout period among the same participants, but the intervention period was short (7 days) and the sample size small (n=8). In the study of Soroka et al. (1998)^13^, the follow-up was longer (6 months), but it also had a small sample size (n=9).

The prospective, randomized, controlled trial by Garneata et al. (2016)^11^ was the study with the most participants and conducted for the longest time. Patients in the intervention arm received a very low-protein vegetarian diet (VLPD; 0.3 g protein/kg ideal body/day) supplemented with ketoanalogues of essential amino acids. The authors discuss that the beneficial effects of the vegetarian diet seems to be mediated by the improvement of metabolic complications of advanced CKD, such as nitrogen balance, mineral metabolism disturbances, metabolic acidosis, and inflammation, rather than by an impact in GFR. However, there are also some limitations of the study. The control group received a diet containing 0.6 g protein/kg per day, which is unusual in many parts of the world.

Garneata et al. (2016)^11^ report that the most beneficial effect of very low-protein diet supplemented with ketoacid analogues of essential amino acids may be the reduction of uremic toxins. Ketoacids lack the amino group bound to the carbon of an amino acid, allowing them to be converted to their respective amino acids without providing additional nitrogen. A diet with 0.3 to 0.4 g of protein per kilogram per day that is supplemented with ketoacids and essential amino acids reduces the production of potentially toxic metabolic products, as well as the load of potassium, phosphorus, and possibly sodium, while still providing calcium.

There is evidence that a diet providing 0.3 g/kg/day of plant-based protein associated with a mixture of essential amino acids and ketoanalogues reduced blood glucose levels and endogenous glucose production and improved insulin sensitivity in six patients with CKD stages 4 and 5^24^, corroborating the benefits of a vegetarian diet.

In conclusion, there is limited evidence comparing vegetarian to non- vegetarian diets in adults with CKD in nondialysis treatment, and only one study reported significant effects on GFR. Additional studies are needed to evaluate the benefits of vegetarian diets through large-scale randomized controlled trials for potential inclusion in clinical recommendations for the management of CKD. To achieve a reliable result, it would be interesting to take measures to ensure participants’ adherence to the diet.

### Practical application

The practical application of this paper is to gather studies that evaluate vegetarian diets for the management of CKD. Vegetarian VLPD supplemented with ketoanalogues is an option to improve some important parameters in the evolution of the disease, yet further studies are needed to determine if it is superior to other types of diet.

This research received no specific grants from public, commercial, or not-for-profit funding agencies.
